# Comparative transcriptomics reveals striking similarities between the bovine and feline isolates of *Tritrichomonas foetus*: consequences for *in silico* drug-target identification

**DOI:** 10.1186/1471-2164-15-955

**Published:** 2014-11-05

**Authors:** Victoria Morin-Adeline, Rodrigo Lomas, Denis O’Meally, Colin Stack, Ana Conesa, Jan Šlapeta

**Affiliations:** Faculty of Veterinary Science, University of Sydney, New South Wales, 2006 Australia; Genomics of Gene Expression Lab, Prince Felipe Research Centre, Valencia, Spain; School of Science and Health, University of Western Sydney, Penrith, New South Wales 2751 Australia

## Abstract

**Background:**

Few, if any, protozoan parasites are reported to exhibit extreme organ tropism like the flagellate *Tritrichomonas foetus.* In cattle, *T. foetus* infects the reproductive system causing abortion, whereas the infection in cats results in chronic large bowel diarrhoea. In the absence of a *T. foetus* genome, we utilized a *de novo* approach to assemble the transcriptome of the bovine and feline genotype to identify host-specific adaptations and virulence factors specific to each genotype*.* Furthermore, a subset of orthologs was used to characterize putative druggable targets and expose complications of *in silico* drug target mining in species with indefinite host-ranges.

**Results:**

Illumina RNA-seq reads were assembled into two representative bovine and feline transcriptomes containing 42,363 and 36,559 contigs, respectively. Coding and non-coding regions of the genome libraries revealed striking similarities, with 24,620 shared homolog pairs reduced down to 7,547 coding orthologs between the two genotypes. The transcriptomes were near identical in functional category distribution; with no indication of selective pressure acting on orthologs despite differences in parasite origins/host. Orthologs formed a large proportion of highly expressed transcripts in both genotypes (bovine genotype: 76%, feline genotype: 56%). Mining the libraries for protease virulence factors revealed the cysteine proteases (CP) to be the most common. In total, 483 and 445 bovine and feline *T. foetus* transcripts were identified as putative proteases based on MEROPS database, with 9 hits to putative protease inhibitors. In bovine *T. foetus,* CP8 is the preferentially transcribed CP while in the feline genotype, transcription of CP7 showed higher abundance. *In silico* druggability analysis of the two genotypes revealed that when host sequences are taken into account, drug targets are genotype-specific.

**Conclusion:**

Gene discovery analysis based on RNA-seq data analysis revealed prominent similarities between the bovine and feline *T. foetus,* suggesting recent adaptation to their respective host/niche. *T. foetus* represents a unique case of a mammalian protozoan expanding its parasitic grasp across distantly related host lineages. Consequences of the host-range for *in silico* drug targeting are exposed here, demonstrating that targets of the parasite in one host are not necessarily ideal for the same parasite in another host.

**Electronic supplementary material:**

The online version of this article (doi:10.1186/1471-2164-15-955) contains supplementary material, which is available to authorized users.

## Background

The protozoan flagellate *Tritrichomonas foetus* belongs to the phylum Parabasalia, which includes the human parasite; *Trichomonas vaginalis* [1] *.* Originally described as a nasal and gastrointestinal commensal of pigs, *T. foetus* infects the urogenital tract of cattle resulting in disease known as trichomoniasis [2–4]. Transmission of the disease to female cows occurs during coitus with infected bulls, which can result in abortion of the foetus [5–8]. Recently, *T. foetus* has been identified as the etiological agent of gastrointestinal infection of domestic cats, where infection results in chronic large bowel diarrhoea [9–11]. The disease in both hosts is very difficult to treat. Bovine trichomoniasis is currently untreatable and the only control measure available to farmers is to cull infected bulls or remove them from the breeding herd. Although treatment options for feline trichomoniasis do exist, they are becoming increasingly ineffective due to evolving parasite drug resistance and issues associated with host drug toxicity [12–14].

*Tritrichomonas foetus* represents an intriguing model to study host-parasite interactions. There has been much conjecture as to the origins of the bovine and feline isolates. In particular, are they different parasites or merely closely related genotypes? Given that both infections are caused by *T. foetus,* it is not surprising that historically they have been assumed to be the same parasite. The adaptation of parasites to different hosts is nothing new, however, the extreme host-organ tropism of the bovine isolate (vagina) and feline isolate (gastrointestinal tract) suggest that they are distinct genotypes that, over the course of their respective evolutions, have preferentially adapted to their respective host/niche [4, 15]. Further evidence as to their distinctiveness (albeit limited) has been demonstrated experimentally when the bovine genotype is introduced into the feline intestinal tract, and vice versa. Although both genotypes are capable of establishing infection in their non-typical host, the pathology is markedly less than on their preferred hosts [10, 16]. Successful delineation of the genotypes would enable a more precise estimation of trichomonas species richness and permit a better epidemiological understanding of the pathological basis of these diseases. Currently, artificial insemination and culling of infected animals ensures that a low infection level is maintained in intensively-managed cattle industries [17, 18]. Infections, however, are still prevalent in extensive farming systems [17, 18]. Moreover, current evaluation of the infection in domestic cats indicated a high prevalence, especially in young (6–12 month old) pedigree catteries [19].

Although it has recently been established that the porcine *T. foetus* (formerly known as *T. suis*) and the bovine *T. foetus* are synonymous [2, 4], the relationship between the bovine and feline *T. foetus* is proving harder to elucidate. Evidence of the limited genetic distinctness between the bovine and the feline isolates is apparent when highly conserved species-level nucleotide sequences for the internal transcribed spacer 2 (ITS2) and elongation factor 1 alpha (EF-1α) of the two genotypes are compared [4, 15, 20]. As the original diagnostic marker, a single nucleotide polymorphism in ITS2 between the bovine and feline *T. foetus* amounts to a sequence difference of only 0.3% [15, 20]. More recent studies demonstrated genetic difference between the two genotypes by analysing the cysteine protease multigene family [4, 21]. This family of genes is known to play a key role in parasite virulence [22–25]. It has been suggested, however, that these minor sequence differences between genotypes may merely represent intraspecific variation and not have any significant phenotypic consequences [26]. Nevertheless, ambiguity arises when single gene assays are used in an attempt to compare very closely related genotypes with broad host ranges, such as the bovine and feline *T. foetus*.

There is the need for a more comprehensive cell-wide approach to enable further elucidation of the relationship between the bovine and feline *T. foetus* genotypes. This is further confounded by a lack of *T. foetus* genome data which has undoubtedly hindered our understanding of host-switching and search for novel drug targets in these parasites. Therefore, in the absence of a genome, we have used RNA-seq to sequence the transcriptome of bovine and feline *T. foetus* genotypes in an attempt to provide a blueprint of functional capacity of each of the host/niche adapted *T. foetus* genotypes. This study represents the first cell-wide comparative analysis of *T. foetus* genotypes, enabling us to determine the extent to which differences between host/niche is reflected in their transcriptomes. In addition to investigating *T. foetus* host-specific biological and virulence mechanisms*,* we utilised our transcriptomic libraries to explore the usefulness of *in silico* techniques for the identification of potential parasite drug targets, taking into account their expanded host-range.

## Results

### Transcriptome

#### Illumina sequencing and transcriptome assembly

A total of 64,744,882 and 64,009,804 100 bp paired-end Illumina reads were obtained following the sequencing of total RNA isolated from bovine and feline *Tritrichomonas foetus* isolates, respectively. Assessment of read quality by FastQC revealed good quality reads (data not shown). Raw reads were mapped onto a small, previously published bovine *T. foetus* EST library and visually assessed to confirm a non-biased and even distribution of sequenced reads. Paired-end sequencing reads from each genotype were assembled using Trinity [27] into two transcriptomes consisting of 42,363 and 36,559 contigs representing the bovine and feline genotypes respectively (Table 1). A mean contig length of 895.2 bp was obtained in the bovine *T. foetus* transcriptome, with minimum and maximum contig lengths of 201 bp and 14,314 bp respectively. The feline *T. foetus* transcriptome had a mean contig length of 806.6 bp with a minimum length of 201 bp and a maximum of 17,195 bp in length.

**Table 1:** **Summary of sequenced reads and the assembled transcriptomes**

Feature	Bovine***T. foetus***	Feline***T. foetus***
Total number of reads	64744882	64009804
Total base pairs (bp)	6539233082	6464990204
Average read length (bp)	101	101
Total number of contigs	42363	36559
Total assembled bases	37882427	29525551
Mean length of contigs (bp)	895.25	806.61
Median contig length (bp)	653	562
% GC content in transcriptome	34.62	34.87
Minimum contig length (bp)	201	201
Maximum contig length (bp)	14314	17195
Contig N50	1259	1178

#### Ortholog identification

**Figure 1:**
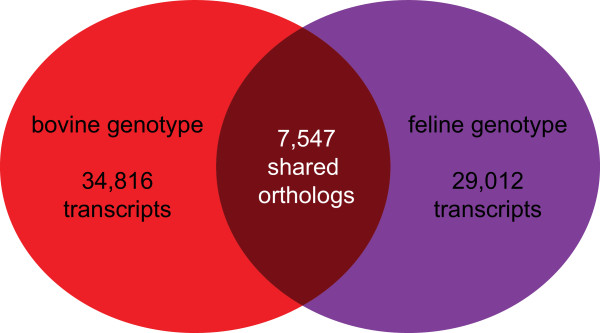
**Distribution of shared transcripts between the bovine and feline** ***T. foetus*** **genotype.** Venn diagram illustrating the shared bovine and feline transcriptome obtained by *de novo* assembly of Illumina RNA-seq sequenced data. A total of 7,547 transcripts were identified as true orthologs shared between the two genotypes.

Homologous transcript pairs between the bovine and the feline genotypes were identified using a reciprocal blast. This method identified 24,620 pairs of homologous transcripts which were further subjected to a BlastX search against the Swissport database (e-value 1 × 10^6^) to remove putative paralogous genes [28, 29]. Orthologous pairs of coding regions were defined by comparing corresponding sequence pairs originating from each genotype and only those found to have identical top-hit in the BlastX results were selected. A total of 7,547 transcript pairs were selected using this method and were thus considered strong orthologs of the bovine and feline *T. foetus* (Figure 1).

A local version of the full-lengther next (FLN) algorithm [30] was implemented to identify putative coding regions in the orthologs using the invertebrate database, as this resulted in superior protein annotation results. Approximately 4,600 transcripts were protein annotated by the algorithm, of which, 1,511 pairs of ortholog pairs were found to be full length transcripts (i.e. the presence of both a start and stop codon). The coding regions of the 1,511 ortholog pairs were isolated using bash scripting.

#### Functional annotation and highly transcribed genes

**Figure 2:**
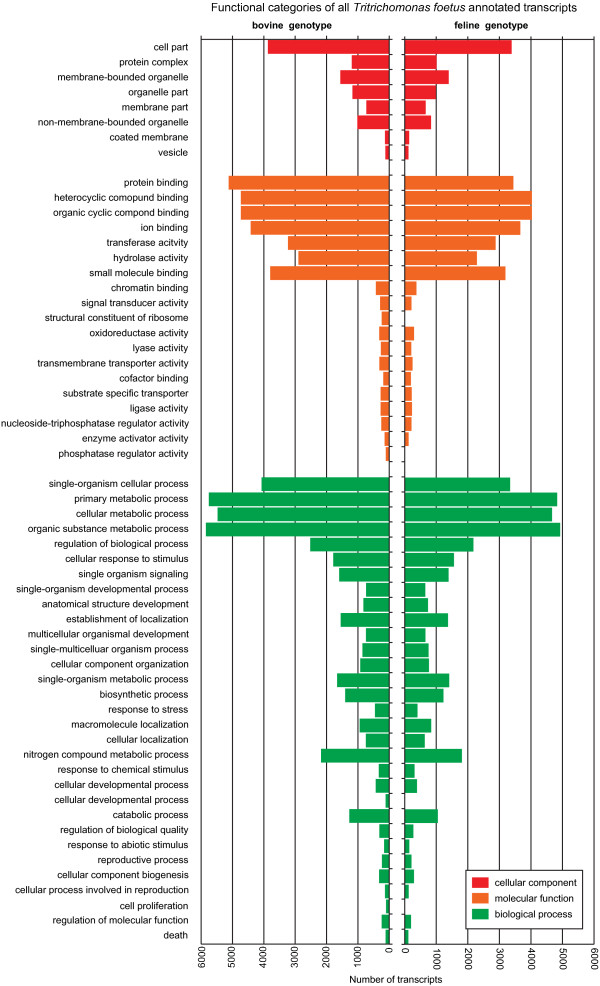
**Top ranked GO categories of the bovine and feline** ***Tritrichomonas foetus*** **whole transcriptomes.** Functional characterisation of the bovine (left) and feline (right) expressed genome based on Gene Ontology categories showing top ranked categories for cellular component, molecular function and biological process. Categories presented represent a minimum threshold filter value of 100 sequences.

**Figure 3:**
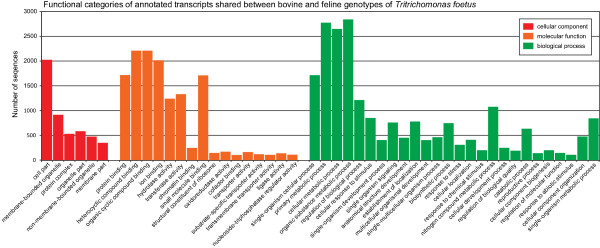
**Representative functional annotation of shared orthologs between the bovine and feline** ***T. foetus*** **genotypes.** Representative Gene Ontology functional categories of the bovine and feline shared orthologous gene pairs for cellular component, molecular function and biological process.

Functional annotation at the BlastX and Gene Ontology level of the assembled transcriptomes were carried out using a combination of locally implemented BlastX searches and Blast2GO [28, 31]. Whole transcriptome annotations and annotations of only orthologs revealed a similar distribution of functional categories between the bovine and feline genotypes (Figures 2, 3). The difference in size of the two assembled transcriptomes can account for the slight variation in the number of transcripts obtained per functional category. The absence of four functional categories from the feline genotype that were identified in the bovine genotype is solely a limitation of the threshold of reported sequences set for presenting GO categories.

**Figure 4:**
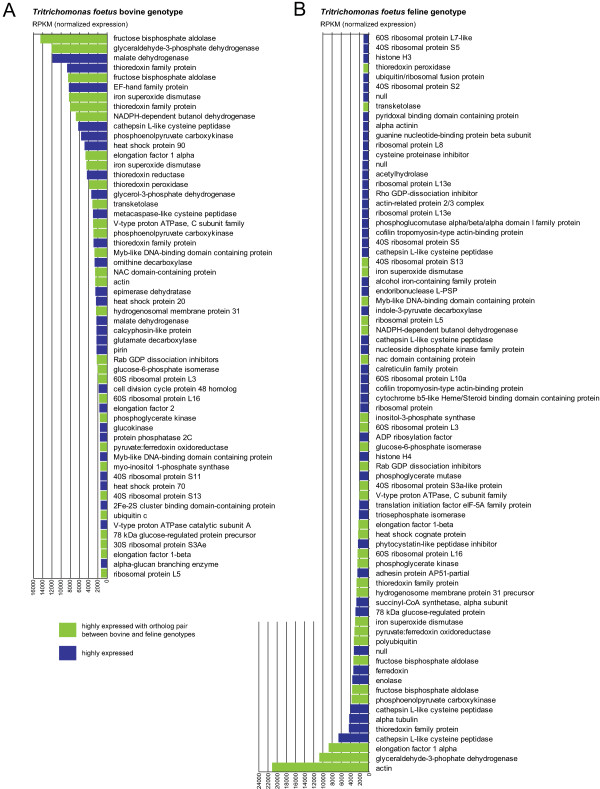
**Annotation of the most highly expressed ortholog genes in the bovine and feline** ***T. foetus.***BlastX functional annotation of ortholog transcripts present within the top 100 highly expressed transcripts in the bovine (left, **A**) and feline (right, **B**) *T. foetus* genotypes after RPKM normalization of reads counts. The graphs show the 76 and 56 orthologous transcripts of the bovine and feline genotypes, respectively. Non-orthologous transcripts are not shown. Green bars represent the orthologs pairs that are highly expressed in both genotypes.

**Figure 5:**
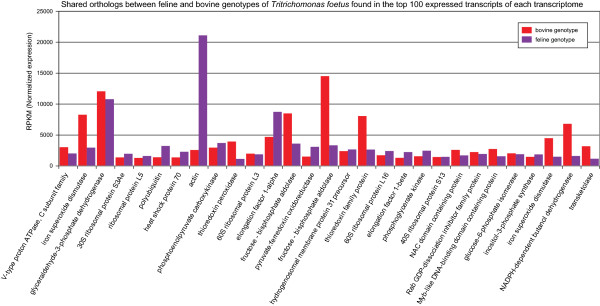
**Normalized expression values of 29 highly expressed orthologous transcripts.** Normalized read counts (RPKM) and BlastX annotation of 29 pairs of orthologous transcripts present within the top 100 highly expressed transcripts in the bovine and feline *T. foetus* genotypes.

To identify the top 100 transcribed genes from each transcriptome, raw sequencing reads were mapped back onto the assembled transcriptome of each genotype and counts were normalized to RPKM (reads per kilobase per million of mapped reads). The top 100 transcripts with the highest RPKM values were selected from the bovine and feline transcriptomes. The top 100 RKPM values for the bovine genotype ranged from 21,107 to 1,119, whereas the RPKM range for the top 100 transcribed genes in the feline transcriptome was from 18,670 to 1,277. Blast annotations obtained previously were used to extract the putative functions of the top 100 bovine and feline transcripts. Within this list, 56 feline transcripts and 76 bovine transcripts were identified as ortholog genes (Figure 4), however, only 29 were orthologs pairs common to both genotypes (Figure 5). The common ortholog genes included mainly genes involved in metabolic activity, oxygen scavenging and regulation of homeostasis; all of which are expected in trichomonads. The remaining 24 and 44 non-ortholog, but highly-expressed transcripts from the bovine and feline genotypes were annotated as ribosomal-related proteins (data not shown).

#### UTR analysis

**Figure 6:**
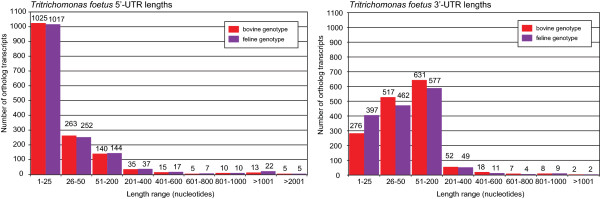
**Length comparisons of untranslated regions (UTR) between the bovine and feline** ***T. foetus*** **.** The number of transcripts from the 1,511 full-length bovine and feline orthologous transcripts with lengths (in nucleotides) of the 5′UTR (left) and 3′UTR (right) falling within the defined ranges.

Un-translated regions (UTRs) of mRNA flank either end of the coding region and contain regulatory elements that dictate translation of genes [32–34]. Only the 1,511 full-length ortholog transcripts were used to compare the UTR lengths and regulatory content of the two *T. foetus* genotypes. The average length of 5′UTRs were 64.2 and 73.3 nucleotides (nt) for the bovine and feline genotypes, respectively. The longest bovine and feline 5′UTRs were 4,325 nt and 4,253 nt in length. The 1 – 25 nt length range contained approximately 67% of 5′UTRs from both genotypes (Figure 6)*.* Longer 5′UTRs (>1,000 nt) were more prominent in the feline genotype with an additional nine feline sequences within this length category compared to that of the bovine genotype. Length analysis of 3′UTRs revealed that 41.8% and 38.2% of bovine and feline sequences, respectively, were found within the 51 – 200 nt length range. On average, 3′UTRs were longer than 5′UTRs with the mean length amounting to 77.7 nt for the bovine *T. foetus* and 70 nt for the feline genotype. The maximum length of 3′UTRs were, however, shorter than the longest 5′UTR with the lengths reaching to 1,360 nt and 1,331 nt for the bovine and feline genotypes, respectively.

**Figure 7:**
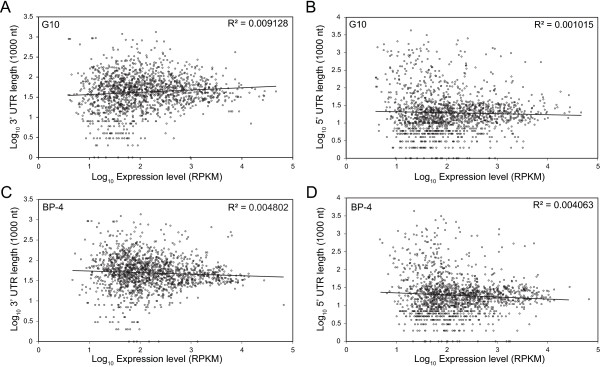
**Correlation between the length of UTRs and the normalized transcript expression (RPKM) for 1,511 shared ortholog.** A non-linear, weighted sum of least square regression model was fitted to plots of the Log_10_ 3′UTR and 5′UTR lengths against the Log10 of normalized expression counts (RPKM) of the respective transcript for the bovine and feline *T. foetus* genotypes. Regression values (R^2^) are quote on the individual plots. **A-B**. 3′UTR and 5′UTR plots respectively, for the feline *T. foetus* genotype. **C-D**. 3′UTR and 5′UTR plots respectively, for the bovine *T. foetus* genotypes.

To identify any patterns of correlation between the 3′UTR and 5′UTR length and the normalized expression count (RPKM), the 1,511 orthologs were plotted in a scatter plot (Figure 7). A non-linear model of regression was used to calculate a weighted R^2^ which takes into account the uneven variance between points across the graph, ensuring that all points contributed equally. In general, R^2^ < 0.01, indicated no correlation between the transcript expression and the length of the UTRs.

**Figure 8:**
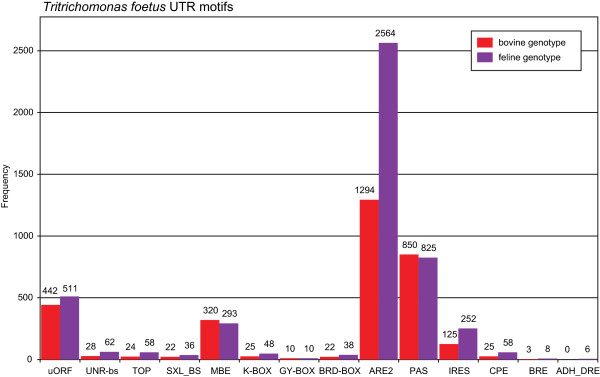
**Frequency of regulatory motifs within the untranslated regions (UTR) of full-length orthologous transcripts.** Frequency of hits to UTR motifs obtained through UTRscan searches of 1,511 full-length orthologous bovine and feline transcripts against the UTRsite of regulatory motifs. Actual number of hits is presented above each bar. Abbreviations: uORF; upstream open reading frame, UNR-bs; UNR binding site, TOP; terminal oligopyrimidine tract, SXL_BS; SXL bind site, MBE; Musashi binding element, K-Box; K-Box, GY-Box; GY-Box, BRD-Box; BRD-Box, ARE2; AU-rich element, PAS; polyadenylation signal, IRES; internal ribosome entry site, CPE; cytoplasmic polyadenylation element, BRE; Bruno responsive element, ADH_DRE; alcohol dehydrogenase down-regulation control element.

Using the UTRscan algorithm [35] to search the UTRsite database [36] for known UTR regulatory motifs, a list of putative motifs were obtained for the two *T. foetus* genotypes. Overall, 14 different motifs were annotated in the UTR regions of 1,511 full-length orthologs between the two genotypes (Figure 8).

All motif patterns in the UTRs were common to both genotypes except for an alcohol dehydrogenase element associated with the 3′UTR of the feline genotype (ADH_DRE) (Figure 8). The most common motif in both genotypes was annotated to the AU-rich class-2 element (ARE2). This amounted to 28.2% and 30.6% of the number of unique hits in the bovine and feline genotypes, respectively. Polyadenylation signals (PAS) were found in 37.2% and 30.5% of the bovine and feline unique hits obtained.

#### Discovery of new proteases and protease inhibitors

Being a strict extracellular parasite, the ability of *T. foetus* parasites to attach to host cells is an essential prerequisite for the initiation and maintenance of infection [37]. During infection, the bovine host mounts a humoral defence against *T. foetus*, however, it is not necessarily sufficient to clear the infection [38]. The secretion of cysteine proteases (CPs) is thought to be an important facet of *T. foetus* virulence. CPs have been demonstrated to cleave and inactivate host protective antibodies, enabling the parasite to remain within the host [39]. To date, there have been 21 CPs identified in the bovine genotype, while only 8 CPs have been identified within the feline genotype [4, 21, 40]. Blast annotation identified a total of 665 and 623 hits to known proteases in the bovine and feline *T. foetus* transcriptomes, respectively. Both transcriptomes were also found to contain 11 hits for protease inhibitors. A total of 389 and 346 CPs with a corresponding 3 and 2 CP inhibitors were identified as belonging to the bovine and feline *T. foetus* respectively.

The full list of proteases was used in a search against the MEROPS peptidase database enabling the further identification of true proteases [41]. Of the initial list obtained through NCBI blast, only 483 and 445 bovine and feline *T. foetus* transcripts received hits from the MEROPS database. Of these hits, 243 bovine transcripts produced hits to a single protease active site, compared to 253 feline transcripts with unique hits. Some sequences obtained hits to multiple active site domains. A total number of 539 bovine and 498 feline active sites were obtained when the results were collapsed to show only unique types of protease active sites per transcript (Table 2). The largest group detected was the cysteine proteases active site architecture, amounting to 52.8% in bovine *T. foetus* and 50% in feline *T. foetus* of the total hits obtained*.* No hits were obtained for glutamic or asparagine proteases in either parasite transcriptomes. Similarly, the same pipeline carried out on *Trichomonas vaginalis* coding genes based on the draft genome produced 475 annotated “protease/peptidase/proteinase” related genes [42]. This comparably corresponded to 221 confirmed putative proteases possessing cysteine-specific active sites in *T. vaginalis* found in the MEROPS database (data not shown).

**Table 2:** **Frequency of protease active site present within the bovine and feline transcriptome**

Functional type	Bovine***T. foetus***	Feline***T. foetus***
Cysteine	285	249
Serine	99	95
Glutamic	0	0
Metallo	88	88
Threonine	22	22
Aspartic	9	9
Inhibitors	35	34
Asparagine	0	0
Mixed	0	0
Unknown	1	1
Overall	539	498

Two protease active sites were unique to either one of the parasite genotypes. In the bovine *T. foetus,* transcript Bc12_comp23753_c0_seq1 produced a hit to the serine active site S51 of the PC clan (MEROPS accession: MER001335) which was not present in the feline *T. foetus* protease list. Conversely, the feline transcript G10_comp5459_c0_seq1 produced a unique hit to the metalloprotease active site M20X of the MH clan (MEROPS Accession: MER001266) which was unmatched in the bovine *T. foetus* protease list.

Raw reads were mapped back onto the putative protease and counts were normalized using RPKM revealing 148 bovine and 113 feline *T. foetus* proteases being expressed at an RPKM of 500 or greater. This RPKM threshold was chosen to signify high expression of proteases. Of the highly expressed proteases, 42.3% of bovine and 39.9% of feline transcripts contained CP active sites, with the MEROPS C01A protease family represented in 65.08% and 40.47% of bovine and feline highly expressed CPs, respectively (Table 3, Additional file 1: Table S1 and Additional file 2: Table S2). The putative *T. foetus* CP sequences were aligned to previously published CP sequences to identify known CPs within our list [4, 40, 43]. Of the 20 bovine and 8 feline published CP sequences, 15 known bovine CPs were among the highly expressed proteases (RPKM >500), whereas CP7 and CP8 were the only known feline CPs with an RPKM above 500 in the feline transcriptome. Interestingly, while CP8 was the most transcribed protease in the bovine genotype, CP7 was found to be highly transcribed in the feline genotype.

**Table 3:** **Type distribution of highly expressed protease in bovine and feline** ***T. foetus***

Protease type	Bovine***T. foetus***	Feline***T. foetus***
Cysteine	63	42
Metallo	34	26
Serine	26	16
Threonine	21	19
Aspartic	4	4
Total	148	113

Of the 11 inhibitors initially found in the BlastX transcriptome annotation, 9 feline *T. foetus* transcripts obtained a positive hit to an inhibitor active site from the MEROPS database, compared to only 8 bovine *T. foetus* transcripts with known inhibitor active site hits. Using BlastN for pairwise alignments of the bovine and feline inhibitor sequences, all bovine sequences align to a feline sequence with approx. 99% identity (Table 4). One putative feline inhibitor sequence (G10_comp9648_c0_seq1) did not align to any bovine sequences and produced a match to the MEROPS I04 protease family (MEROPS accession: MER003981).

**Table 4:** **Summary of aligned protease inhibitors and their predicted MEROPS family**

Feline transcript	Bovine transcript	Alignment	Identity	Predicted protease family	MEROPS accession number
G10_comp7804_c0_seq1	Bc12_comp9941_c0_seq1	333/334	99%	I25B	MER018186
G10_comp2876_c0_seq1	Bc12_comp7451_c0_seq2	936/943	99%	I04	MER018805, MER018695, MER023786 (bovine only)
G10_comp7790_c0_seq1	Bc12_comp7451_c0_seq1	1139/1145	99%	I04	MER018805, MER016306 (feline only), MER018807 (feline only), MER027490 (feline only), MER018695 (bovine only)
G10_comp7405_c0_seq1	Bc12_comp9915_c0_seq1	293/297	99%	I25B	MER018937, MER018172 (bovine only)
G10_comp3687_c0_seq1	Bc12_comp3242_c0_seq1	311/314	99%	I25B	MER018186
G10_comp18864_c0_seq1	Bc12_comp5569_c0_seq1	394/401	98%	I04	MER018805 (feline only), MER003223 (feline only), MER018696 (bovine only), MER016306 (bovine only)
G10_comp17054_c0_seq1	Bc12_comp4109_c0_seq1	899/903	99%	I04	MER018698, MER023885 (bovine only)
G10_comp12847_c0_seq1	Bc12_comp13520_c0_seq1	339/342	99%	I25B	MER181466, MER166026

#### Analysis of sequence divergence

**Figure 9:**
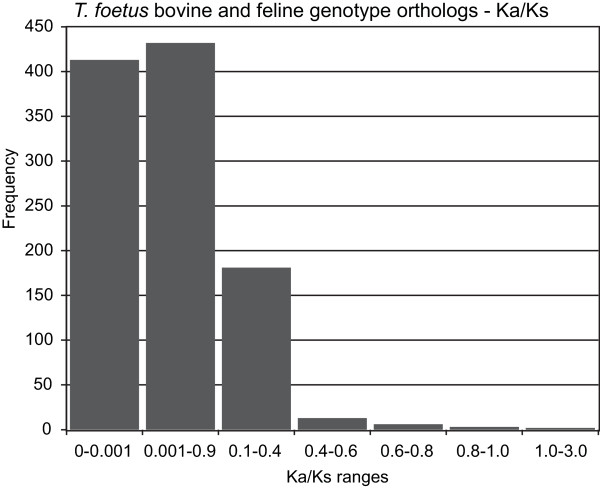
**Frequency of Ka/Ks values for full-length orthologous transcripts between the bovine and feline genotype.** Frequency of orthologous transcript pairs producing Ka/Ks values within various ranges.

Pairwise codon-alignments of the 1,511 full-length ortholog transcripts shared between the bovine and feline *T. foetus,* revealed that only 1,050 transcript pairs (69.5%) were sufficiently divergent to allow for Ka/Ks calculation. The aligned pairs of orthologs showed an average substitution of 10.6 nt ranging from a minimum of 4 nt to a maximum of 167 nt substitutions. While strong sequence conservation (Ka/Ks: <0.1) was detected in 80.41% of the coding orthologs analysed (Figure 9), weak purifying selection, that is, a low rate of protein change denoted by a Ka/Ks ratio within the 0.5 – 1.0 range was demonstrated by 14 orthologs pairs. A single ortholog pair had a Ka/Ks ratio of approximately 1, signifying neutral selection (no selective pressure), while only one other pair showed gene divergence with a Ka/Ks ratio greater than 1.

Comparison of Gene Ontology (GO) terms between highly conserved, less-conserved and the divergent group of orthologs showed that “binding activity” is over-represented in all 3 groups. This functional category is represented by 66.5%, 50%, and 100% of orthologs belonging to the high purifying, weak purifying and the divergent ortholog set, respectively. Within the high purifying group (Ka/Ks <0.5), 211 sequences were specifically involved in ATP binding activity, while 13.5% of transcripts were metabolically active transcripts. The divergent group of orthologs presented hits to an unknown protein containing a Myb-like binding domain (GO: 0003677, GO:0003682).

Other GO categories that were obtained included “Transport activity” and “Translational activity” which were represented by 20% and 10% orthologs from the weak purifying subset, respectively.

#### Druggability

To explore *in silico* drug targeting pipelines between *T. foetus* genotypes*,* only the 1,511 predicted full-length orthologs were used for identification of potential drug target, with an added level of search stringency aimed at identifying non-host targets. Of the 1,511 ortholog pairs of transcripts blasted to their respective host proteome, 123 bovine *T. foetus* and 105 feline *T. foetus* transcripts were found to be unique to the parasite (i.e. not found in their respective hosts). Approximately half of the parasite-only transcripts obtained (bovine: 48/105; feline: 59/123) produced positive results to one or more druggable domains. From the feline BlastX results to druggable domains, 49.1% (416/846) of the domains adhere to the Lipinsky rule of 5 for small molecule binding. This number was lowered to 43% (184/424) in the bovine druggable transcripts (Additional file 3: Table S3 and Additional file 4: Table S4).

## Discussion

In this study, we characterized draft transcriptomes of two genotypes of *Tritrichomonas foetus;* a trichomonad of veterinary significance, secondary in prominence only to the human *Trichomonas vaginalis*. In the absence of a genome or sufficient background proteomics, a *de novo* RNA-seq approach was used as an economical and high-throughput cell-wide gene discovery technique. Currently, only a small expressed sequence library of the bovine *T. foetus* is available in the public domain [40]. Here, existing expressed sequence data of the bovine *T. foetus* is augmented and we leverage the field by providing the first comprehensive expressed sequence library of a feline *T. foetus* genotype*.* Gene discovery via RNA sequencing projects provide an accurate representation of transcriptionally active regions of the genome, thus proving an accurate starting point for the unearthing of undiscovered genes [44, 45]. With the expression blueprints of the bovine and feline *T. foetus* genotypes, the first cell-wide comparison of shared genes was undertaken and an *in silico* novel drug target analysis was explored. A draft genome has previously been published for the related human pathogen *Trichomonas vaginalis* [42] *.* However, phylogenetically, *T. vaginalis* and *T. foetus* are significantly divergent, impeding extension of molecular information between the two parasites*.* Hence the sequence library we have constructed is currently the best depiction of the *T. foetus* genome to date and will reinforce the platform for future experimental studies on *T. foetus* cell biology and host-parasite interactions.

In general, the two *T. foetus* transcriptomes are comparable in size with a near identical GC content of approximately 35%. While this is equivalent to the GC content of *T. vaginalis,* it is lower than the previously published 41.9% for bovine *T. foetus,* presumably owing to small library-size bias of the latter [40, 42] *.* Given the very distinct fundamental host requirements and the vastly different host niches occupied by these two genotypes, we were interested to identify to what degree this would be reflected in their respective gene profiles. We found no biologically significant differences in the distribution of functional annotations between the two *T. foetus* transcriptomes, despite the slight variances in culture conditions of the two genotypes in this study. This suggests that the two genotypes possess remarkably similar basal functioning. Resemblances in functional capacity in transcriptome-wide studies of other protozoan groups are not uncommon [46, 47]. To our knowledge, this has not been documented in flagellate protozoans extending over a diverse host range such as *T. foetus*. Limited hints to host-origin were observed as approximately half of the transcripts were flagged as homologs between the genotypes, with 30% being orthologs shared from a common ancestor. As an alternative method of comparing the two *T. foetus* genotypes, the 100 most highly transcribed genes from each transcriptome were identified through counts of uniquely-mapped sequencing reads. Unsurprisingly, functional categories of highly expressed *T. foetus* genes included nutrition-related genes, transcription factors and oxygen scavenging genes, with over 50% of the sequences being identified as shared orthologs of the two genotypes. Although minor loss of detail is imminent, discarding multi-mapped reads for expression counts has shown overall reliability in depiction of highly expressed profiles from RNA-seq data [45, 48]. Of the comparable highly transcribed orthologs, the most notable was the 8-fold difference in actin expression between the two isolates. Actin is associated with a myriad of functions including whole cell and intracellular vacuole movement that contribute to parasite virulence [49–52]. Changes in cell morphology and increased interaction with host cells is associated with differential expression of actin in *T. vaginalis* [53]. While potential culture artefacts cannot be ruled out in the current study, the discrepancy of actin expression between the genotypes is worthy of further characterization to better understand *T. foetus* virulence.

Within their respective host niches, the bovine and feline *T. foetus* genotypes are exposed to extreme environmental constraints that place genes under selective pressure as an adaptive mechanism. These responses are seen in sequences as a ratio of synonymous to non-synonymous substitutions (Ka/Ks), which relates to the ratio of silent mutations to amino-acid changing mutations likely to alter protein functionality [54, 55]. Positive selective change was apparent in two pairs of shared *T. foetus* orthologs both, producing hits to a Myb DNA-binding domain containing protein. As one of the largest families of transcription factors, Myb domain-containing proteins act to regulate the transcription of genes that control and implement important biological processes such as growth, encystation and virulence [43, 56, 57]. Strong divergence of *T. foetus* transcription factors could imply unique adjustments of gene expression between the two genotypes according to their hosts*.* A total of 445 and 461 bovine and feline Myb-like proteins, respectively, were annotated in the transcriptomes, suggesting an important role for these transcription factors*.* In the related *T. vaginalis,* Myb-like nuclear proteins act to regulate transcription of a gene family that encode surface cytoadhesives such as the AP65 protein essential for the parasite adherence to host cells [58–61]. Taken together, the near identical transcriptomes and diversification of certain transcription factors suggest that transcription and post-transcription regulation form a major aspect of phenotypic host-specificity in *T. foetus*. It would seem that perhaps the pressure imposed by the differing hosts/niche environments has not, as of yet, been sufficient to drive molecular diversification at the amino acid level between these two genotypes. Alternatively, these may not be evident in the absence of specific cues for the host. The Myb-transcription factors present as ideal candidates for initial investigations into the extreme versatility of *T. foetus* that allows it to adapt rapidly to new, extreme environments presented by their non-original host.

Untranslated regions (UTRs) flanking either end of mRNA coding regions contain inherent information, such as motif sequences, that govern and regulate the downstream translation of a protein [32, 33]. Under varying environmental conditions, UTRs have the capacity to permit instantaneous phenotypic changes within the parasite to permit rapid responses to biological and environmental cues [32–34]. Here, we mined the untranslated regions (UTRs) for translation regulatory features that may be acting in the bovine and feline *T. foetus* genotypes. One such feature is the length of the UTRs which has been associated with mRNA stability and translational efficiency [62]. A compilation of UTR lengths from UTR databases indicates that across groups of living organisms, 3′UTR are longer than 5′UTRs [63]. Indeed the mean length of *T. foetus* UTRs in this study follows the same length bias*.* The validity of comparing UTR length to determine organism complexity, expression levels and breadth has generated mixed results [64–67]. In the closely related *T. vaginalis,* the length of the glycolytic glyceraldehydes-2-phosphate dehydrogenase 3′UTR did not show correlation to expression of the enzyme [65]. A more tangible manner of UTR translational regulation is mediated through binding of small mRNA-binding protein to cis-elements in the UTR region of the target protein [34]. Annotation of 5′ and 3′ UTRs to known patterns in this study provide a glimpse of putative regulatory motifs at play in *T. foetus*. Common motifs such as up-stream open reading frames (uORF) [68], internal ribosomal entry sites (IRES) [69] and AU-rich class 2 elements (ARE2) [70] have been identified here in the UTRs of both *T. foetus* genotypes. Functionality of several of the motifs found in *T. foetus* have been described in protozoan and in fact, in *Plasmodium*, uORF presents an interesting case as it regulates a virulence-associated trait according to host physiological factors [71]. The unique feline *T. foetus* genotype motif; ADH-DRE, has not been described in protozoans and is related to the down-regulation of the alcohol dehydrogenase gene [72, 73]. Nevertheless, finding common motif matches between *T. foetus* and other organisms in public databases confirms that the conserved nature of motif patterns extend to *T. foetus*. Here, we attempted to by-pass the inherent transcription level limitation of RNA-seq to provide an overview of putative translation-related mechanisms in *T. foetus.* Bioinformatics tools, however, are currently relatively un-reliable in determining true functional regulatory motifs [74]. Experimental characterisation of these motifs in *T. foetus* is required to elucidate regulatory relationships between regulators and the target in these parasites*.*

Proteases are expressed by all organisms, playing a critical role in protein catabolism. In parasites, however, these enzymes have evolved specialized functions and are directly involved in numerous host-parasite interactions. Proteases, also known as peptidases, can be classified into seven functional categories based on the principal residue occupying the active site: Aspartic, Cysteine, Glutamic, Serine, Threonine, Metallo and Mixed [41, 75, 76]. Each of these can be further divided into clans and families. Trichomonad parasites also release soluble proteases *in vitro* and *in vivo;* the best studied of these being the cysteine proteases (CP). Proteases released into the host milieu, as well as those on the surface of parasites, are important virulence factors involved in host-cell adherence, evasion of host immunity and host cell cytotoxicity [23–25, 39]. Approximately half of the proteases found in the transcriptomes in this study contained cysteine active sites and these were over-represented within a subset of highly expressed proteases. In bovine *T. foetus,* the most dominant CP family expressed was a papain type CP of the CA clan (C01A); a large family of CPs involved in facilitating infection in protozoa (reviewed in: [22]). This family is slightly less represented in the highest expressed proteases of the feline *T. foetus.* Regulation of the type of CP secreted by parasites has important implications for the extent of infection in the host. In the related *T. vaginalis,* secreted CP fraction has been demonstrated to promote host-cell apoptosis. Host-cell specificity of CPs, however, is apparent when incubation of *T. vaginalis* CP30 with non-host bovine cells fails to induce the same level of cellular destruction compared to the effect of bovine *T. foetus* CP8 on bovine cells [77, 78]. Here, we confirm that CP8 is the most transcribed CP in the bovine *T. foetus* as reported by Huang *et al*. [40]. To the contrary, CP7 was found to be more transcribed than CP8 in feline *T. foetus* suggesting that the increased expression of CP7 in feline genotype is a host-specific adapted virulence trait. CPs are capable of inducing varying levels of cellular destruction depending on cell source and type. The difference in the major type of CP expressed between the genotypes may explain the slightly varied pathology described in experimental cross-infection of the hosts but the significance of this has yet to be elucidated [10, 16] *.* Our analysis revealed that the bovine transcriptome contained more hits to proteases than the feline *T. foetus* transcriptome which could be due to the fractionally higher sequence reads obtained for the bovine *T. foetus*. The significance of this finding will require full genome sequencing and analysis.

The collection of expressed sequences from the bovine and feline *T. foetus* genotypes made available in this study presents an opportunity for low-cost *in silico* mining of novel drug targets worthy of experimental follow-up. With mounting reports of drug resistance and toxic host side-effects, the treatment of choice for human and feline trichomonad infections remains the 5-nitromidazoles drugs [12, 13, 79]. To date, significant, yet non-model protozoan species have been overlooked for *in silico* mining for druggable targets. Computational pipelines for drug-target discovery have been limited to the few high-profile protozoans with a sequenced genome or focused on identifying druggable features at the host-parasite interface [52, 80, 81]. Here we explore drug target identification for a unique case of a protozoan species with a broad, distantly-related host-range. Novel drug targets for experimental follow-up have to be compatible and non-toxic for the host-species undergoing treatment. In our analysis we intentionally only included shared, full-length *T. foetus* sequences to gain insights as to how the presence of endogenous host proteins could affect strategies for drug target identification of the same parasite species. By excluding similar host genes, a maximum of 5% of the druggable parasite-unique genes remained, the majority of which were not common between the two genotypes. While the list we generated from our analysis is purposed to be more illustrative rather than definitive; the findings stress the importance of taking different hosts into account as a part of target prioritization in more generalist parasites like *T. foetus*.

## Conclusions

The expressed genes of the bovine and feline *Tritrichomonas foetus* genotypes offer insights into the breadth of both the *T. foetus* coding and non-coding genomes*.* This parasite represents an interesting biological model as it represents a unique case of a protozoan expanding its parasitic foothold across distantly related mammalian hosts. Despite extreme environmental conditions found between bovine urogenital tract and the feline digestive tract inhabited by the two genotypes, they possess near identical functional category distribution of expressed genes with no indication of molecular-level divergence. This reinforces the fact that taxonomically, the bovine and feline *T. foetus* represent two genotypes displaying intra-specific variation. Host-specific adaptation strategies appear to be focused on post-transcription regulation influenced by environmental cues within the two host niches. In this manner, expression patterns of virulence genes may differ in accordance to their host. Although RNA-seq has provided insights into expression patterns, proteomics studies need to be carried out to examine the full extent of these patterns between the genotypes. Nevertheless, the role of transcriptional and post-transcriptional regulation in *T. foetus* warrants consideration to guide further research since studies on an environmental-dependent gene in one genotype will not necessarily be representative of the other genotype. Hence, host genes and biology have to be taken into account, particularly in the design of new drug strategies. While *in silico* methods offer an ideal starting point for novel drug target identification, here we highlight the importance of taking both genotypes and their hosts into account to avoid downstream mis-identification of common drug targets. Taken as a whole, the cell-wide gene library of the bovine and feline *T. foetus* generated in this study is a useful platform to guide trichomonad research.

## Methods

### Cultures

Two *Tritrichomonas foetus* genotypes were used for this study; a genotype isolated from a bovine host and a feline genotype originating from a feline host. The bovine genotype; *Tritrichomonas foetus* BP-4: Beltsville (ATCC® 30003™, the American Type Culture Collection, Manassas, USA). The feline genotype; *Tritrichomonas foetus* Sydney-G10/I (cryopreserved in the culture collection at the Faculty of Veterinary Science, The University of Sydney) [15]. Both genotypes were axenically maintained at 37°C by 48 hour passages in a trypticase, yeast extract and maltose (TYM)-medium at pH 7.2. The media was supplemented with 10% (v/v) heat-inactivated lamb serum. To ensure adequate growth, media used for the bovine genotype was further supplemented with 0.05% (w/v) bacterial agar. A mixture of PenStrep and Fungizone was added at a final concentration of 100 μg/ml to both media to safeguard against biological contaminant growth.

### Transcriptome

#### Sample preparation and RNA isolation

Bovine and feline *T. foetus* cells at the mid-exponential phase in culture were collected and 1 × 10^7^ cells were pelleted at 3220 × g for 5 min. Cells were resuspended in 600 μl RTL buffer, according to the RNeasy Micro kit (Qiagen) protocol and homogenisation was carried out in a FastPrep® - 24 Instrument (MP Biomedicals, USA) for 30 seconds at 4 m/s. An in-column DNAase (Sigma-Aldrich) treatment step was carried out with an incubation time of 15 minutes at room temperature. RNA was eluted in 30 μl of sterile water and assessed both qualitatively and quantitatively using a 2100 Bioanalyzer (Agilent Technologies, Inc). Samples were then transferred into RNAstable® tubes (Biometrica) and preserved by drying in a Savant SpeedVac concentrator connected to a vapour trap for 1 hour, in accordance with the manufacturer’s instructions. Paired-end RNA sequencing on Illumina HiSeq2000 was performed at Macrogen (Seoul, Korea).

#### Transcriptome sequence analysis and assembly

Raw RNA sequenced reads were subjected to quality control analysis using FastQC (Babraham Bioinformatics). Sequenced reads were mapped onto a small indexed library of published *T. foetus* coding genes using a combination of bowtie (version bowtie/2.1.0) and TopHat (version tophat/2.0.8) and visualized using IGV (version igv/2.3.3-4G) [40, 82, 83]. A *de novo* approach using default parameters in Trinity, according to [27], was adopted for assembly of left and right reads belonging to each genotype individually, resulting in two libraries representing the feline and bovine expressed genome.

#### Ortholog prediction

To obtain a list of coding homologue pairs shared between the two *T. foetus* genotypes, a reciprocal blast was performed using the Galaxy platform with the assembled feline and bovine *T. foetus* transcriptomes [84–86]. Putative paralogues were removed by blasting the homolog pairs against the SwissProt database with an e-value cut-off of < 1 × 10^6^. Top blast hits for each transcript pairs were collected and filtered using bash scripting to retrieve 7547 pairs of transcripts showing identical hits to the same protein, which were subsequently utilized as ortholog pairs.

The full-lengther Next algorithm [30] was implemented on the list of 7547 ortholog pairs using the invertebrate database and default parameters to identify full-length transcripts. Based on the criterion that both transcripts from each pair were full length (i.e. contained both a start and stop codon), 1151 pairs of transcripts were selected for further analysis. For each pair of full-length orthologues, bash scripting was used to isolate all coding regions, 5′UTR and 3′UTRs were extracted for further analysis.

#### Functional annotation and identification of highly expressed genes

The entire assembled transcriptomes and the two lists of 7547 orthologues were annotated through local BlastX searches against the NCBI non-redundant (nr) database abiding to a cut off e-value of 1 × 10^3^ [28]. Gene Ontology (GO) level annotation of the assembled sequences were retrieved using default setting in Blast2GO using the results of the local NCBI BlastX [31]. Combined graphs were generated for each analysis with a level 3 cut-off and a minimum sequence threshold of 100 per category.

To identify the top 100 highly expressed genes between the bovine and feline *T. foetus,* a bash script was written to create .gtf files for each transcriptome. The assembled transcriptomes were indexed using Bowtie (version bowtie/2.1.0) and raw sequenced reads were mapped back onto the assembled transcriptomes using default TopHat (version tophat/2.0.8) settings [82, 83]. Qualimap compute-counts (version qualimap/0.7.1) [87] were subsequently used with the ‘uniquely-mapped-reads’ algorithm to count the number of raw reads that successfully mapped back onto each assembled transcriptome. Counts were normalized to transcript length using RPKM (reads per kilobase per million of reads). BlastX results of transcripts with the top 100 RPKM were extracted from the whole transcriptome blast against the NCBI nr database blast for comparison.

#### UTR extraction and annotation

Upon identification of the 1,511 full-length orthologous transcripts, the 5′ and 3′ UTRs were isolated based on identification of the start and stop codon predicted by the full-lengther Next algorithm [30]. Scripting enabled calculation of the lengths of the UTRs and comparative graphs were created. Scatter plots to compare the length of the 3′UTR and 5′UTRs to the normalized transcript expression counts (RPKM) were created and the sum of least square, straight line regression model was adopted in GraphPad Prime 6 (California, USA). A non-linear weighted R^2^ (weighted by 1/Y^2) was chosen to minimize the sum of the squares of the relative distance of the points from the line. A local version of Patsearch [88] was implemented with the UTRscan algorithm [35] to search for known motif patterns from UTRsite [36]. The UTR regions were extracted and only motifs annotated to within these regions were isolated.

#### Discovery of new proteases and protease inhibitors

The BlastX annotations of both the feline and the bovine transcriptomes were used to search for the synonymous terms; “protease”, “peptidase” and “proteinase”. Positive search hits to any of the terms were extracted creating two genotype-specific lists of predicated proteases. The lists were further mined for the term “inhibitor”, and all positive matches were removed and used to create separate lists of putative protease inhibitors from the bovine and feline *T. foetus. Tritrichomonas foetus* transcript IDs were used to retrieve corresponding nucleotide sequences from their respective assembled transcriptome ending with 665 bovine and 623 feline putative protease transcripts. These transcripts were submitted to the available online batch blasting tool on MEROPS peptidase database to search for similarities to known protease active sites [41, 75]. Similarly, the protease inhibitor sequences were subjected to the online blast search against the MEROPS inhibitor database. The resulting feline and bovine inhibitor nucleotide sequences were compared by pairwise alignment using a 2-sequence BlastN [28]. *Tritrichomonas foetus* transcripts with a positive hit against a MEROPS entry were considered a putative protease. As a comparative control for the proteases, identical searches were implemented on a list of 59,672 annotated *Trichomonas vaginalis* coding genes downloaded from TrichDB [89].

In order to identify which putative sequences in the two transcriptomes match to published *T. foetus* cysteine protease (CP) sequences, a 2-sequence BlastN pairwise nucleotide alignment between the MEROPS-confirmed bovine proteases and published bovine CP sequences from Huang et al. [40] and Šlapeta et al. [4] were carried out. Similarly, the BlastN was carried out between the MEROP-confirmed feline sequences and published feline CP sequences from Šlapeta *et al*. [4]. Qualimap compute-counts with the proportional algorithm was used to count the number of raw sequencing reads that mapped back onto the putative proteases [87]. Resultant expression count values were normalized to transcript length using RPKM and all transcripts with normalized expression values of 500 or greater were selected for comparison between the two genotypes.

#### Analysis of sequence divergence

Pairs of orthologous coding sequences were translated into protein sequences using a local version of TranslatorX and pairwise alignments of each pair were generated using ClustralW2 through an array bash scripting [90]. Pairwise proteins alignments were translated into codon alignments using Pal2Nal (v14) and the perl parseFastaIntoAXT.pl script which is distributed with kaks-calculator converted the resulting alignment file into the required format [91, 92]. The codon alignments were subsequently used to calculate substitution rates for non-synonymous (Ka) and synonymous (Ks) sites using a 14-model averaging method implemented in KaKs_Calculator2.0 [92].

#### Druggability

A host database of bovine protein sequences (the full official gene set v2 protein sequences) was retrieved from BovineGenome.org and the complete peptide database for the domestic cat was retrieved from ENSEMBL (release 6.2.74) [93]. The list of 1,511 ortholog pairs belonging to the bovine and feline *T. foetus* were queried against their respective host protein databases using BlastX (e-value 0.0001) [28]. Parasite transcripts that did not produce a common Blast hit with their respective host were extracted and queried in a further BlastX against known druggable domains retrieved from the ChEMBL’s DrugEBility database (e-value 0.0001) [94]. Positive hits were matched with domain information to identify which transcripts contain domains that satisfy Lipinski ‘rule of 5’ of druggability [95].

### Availability of supporting datasets

The assembled transcriptome libraries supporting the results of this article are available in the LabArchives repository [96], [http://dx.doi.org/10.6070/H4GH9FWD]. All raw sequence read data has been submitted to the sequence read data (SRA) repository under the BioProject accession PRJNA246668 [http://www.ncbi.nlm.nih.gov/bioproject/PRJNA246668].

The additional tables (Additional file 1: Tables S1, Additional file 2: Table S2, Additional file 3: Table S3 and Additional file 4: Table S4) supporting the results of this article are included within the article.

## Electronic supplementary material

Additional file 1: **Highly expressed proteases of the bovine** ***T. foetus*** **genotype.** Two excel documents showing assembled bovine transcript ID (from this study) that presents with a hit to the MEROPs database, in order of the highest estimation of protease expression (RPKM) within the bovine genotype. Transcript length, specific hits to MEROPS proteases family and MEROPS accession numbers are presented. Transcript sequences that successfully produce pairwise alignment to published bovine cysteine proteases are also shown. (XLSX 20 KB)

Additional file 2: **Highly expressed proteases of the feline** ***T. foetus*** **genotype.** Two excel documents showing assembled feline transcript ID (from this study) that presents with a hit to the MEROPs database, in order of the highest estimation of protease expression (RPKM) within the feline genotype. Transcript length, specific hits to MEROPS proteases family and MEROPS accession numbers are presented. Transcript sequences that successfully produce pairwise alignment to published feline cysteine proteases are also shown. (XLSX 41 KB)

Additional file 3: **Non-host, putative bovine** ***T. foetus*** **genotype druggable domains.** Bovine *T. foetus* non-host (i.e. unique to the parasite) transcripts which presented a positive hit to known druggable domains are presented, along with the EMBL DrugGAbility px number (SCOP domain), the associated pdb code and information regarding the druggable domain found. Transcript IDs are repeated in the table as more than one domain could associated within each transcript. Adherence to the Lipinski rule of 5 for druggablity is presented as “Y” for true or “N” for false. BlastX annotation and length of the assembled transcript is also presented. (XLSX 32 KB)

Additional file 4: **Non-host, putative feline** ***T. foetus*** **genotype druggable domains.** Feline *T. foetus* non-host (i.e. unique to the parasite) transcripts which presented a positive hit to known druggable domains are presented, along with the EMBL DrugGAbility px number (SCOP domain), the associated pdb code and information regarding the druggable domain found. Transcript IDs are repeated in the table as more than one domain could be associated within each transcript. Adherence to the Lipinski rule of 5 for druggablity is presented as “Y” for true or “N” for false. BlastX annotation and length of the assembled transcript is also presented. (XLSX 51 KB)
